# Stable Isotope Labeling to Track De Novo Fatty Acid Synthesis in Mouse Cartilage‐Enriched Joint Tissue In Vivo: A Practical Workflow

**DOI:** 10.1002/jor.70290

**Published:** 2026-10-04

**Authors:** Shadi Moradi, Anupama Binoy, Shouan Zhu

**Affiliations:** ^1^ Department of Biomedical Sciences, Heritage College of Osteopathic Medicine (HCOM) Ohio University Athens Ohio USA; ^2^ Ohio Musculoskeletal and Neurological Institute (OMNI), Heritage College of Osteopathic Medicine (HCOM) Ohio University Athens Ohio USA; ^3^ Institute of Molecular Medicine and Aging (IMMA), Heritage College of Osteopathic Medicine (HCOM) Ohio University Athens Ohio USA; ^4^ Diabetes Institute (DI), Heritage College of Osteopathic Medicine (HCOM) Ohio University Athens Ohio USA

**Keywords:** cartilage, de novo lipogenesis, fatty acid synthesis, stable Isotope

## Abstract

Cartilage is traditionally viewed as a metabolically quiescent tissue that relies primarily on exogenous lipid uptake to meet its energetic and structural demands. However, increasing evidence suggests that chondrocytes exhibit metabolically active behaviors, particularly under aging and pathological conditions. Here, we developed and applied an in vivo deuterium oxide (D_2_O)–based stable isotope labeling approach to directly quantify de novo fatty‐acid synthesis in cartilage. Using gas chromatography–mass spectrometry (GC–MS), we tracked deuterium incorporation into palmitate in serum and articular cartilage from wild‐type (WT) and cartilage‐specific acetyl‐CoA carboxylase 1 (ACC1) knockout mice. Progressive incorporation of deuterium into palmitate confirmed active fatty‐acid synthesis in cartilage, with labeling kinetics distinct from those observed in serum, consistent with slower lipid turnover in the matrix‐dense cartilage environment. Although fractional new synthesis of palmitate was comparable between genotypes during the labeling window, ACC1‐deficient cartilage exhibited reduced palmitate abundance and decreased higher‐order isotopomer enrichment at early labeling time points, indicating attenuated lipogenic flux. These findings provide direct in vivo evidence that chondrocytes possess intrinsic lipogenic capacity and that ACC1 contributes to the maintenance of the cartilage lipid pool. Together, this study establishes D_2_O‐based isotope tracing as a robust and quantitative framework for assessing cartilage lipid metabolism in vivo and reveals de novo lipogenesis as an active metabolic process in cartilage.

**Clinical Significance:**

This approach offers a valuable platform for investigating lipid dysregulation in osteoarthritis and other joint diseases.

## 1 Introduction

Obesity is widely recognized as a major contributor to osteoarthritis (OA) [1]. In addition to increasing mechanical load on weight‐bearing joints, excess adiposity induces systemic metabolic disturbances that directly affect joint tissues [2, 3]. Clinical studies demonstrate that reductions in body fat, rather than total body weight, are more closely associated with improvements in OA symptoms, underscoring the importance of metabolic factors in disease progression [4]. Although the relationship between dyslipidemia and OA remains debated [5, 6], accumulating evidence supports a strong association between altered lipid metabolism and joint degeneration [7, 8].

Lipid accumulation, often described as lipotoxicity, has been observed in osteoarthritic cartilage, where chondrocytes exhibit elevated intracellular lipid content that correlates with disease severity. These changes have been linked to dysregulated expression of genes controlling lipid efflux and transport, as well as epigenetic alterations in cholesterol‐regulating enzymes that influence OA progression [9, 10]. Consistent with these observations, animal studies have shown that high‐fat diets accelerate OA development by promoting synovial inflammation, osteophyte formation, systemic metabolic stress, and overall joint deterioration [11, 12].

Under physiological conditions, chondrocytes contain small lipid droplets that serve as an energy reservoir within the avascular cartilage environment. However, aging and metabolic stress are associated with increased lipid deposition in cartilage, and multiple studies have linked lipid accumulation to OA progression [13]. Recent work further demonstrated that lipid abundance in chondrocytes increases in response to stress or inflammatory stimuli, suggesting that cartilage possesses an intrinsic lipid regulatory system distinct from classical adipose tissue metabolism [14].

Alterations in lipid composition may also contribute to OA pathogenesis. Elevated levels of pro‐inflammatory fatty acids, including arachidonic, linoleic, and adrenic acids, have been associated with enhanced cartilage degeneration through eicosanoid‐mediated inflammatory pathways. In contrast, ω‐3 fatty acids tend to decrease, resulting in an increased ω‐6/ω‐3 ratio and a more pro‐inflammatory lipid milieu. Despite these observations, the mechanisms by which obesity reshapes cartilage lipid composition remain incompletely understood [15].

Emerging evidence indicates that articular chondrocytes are metabolically active and capable of importing lipids from their extracellular environment. Fatty acids such as palmitate, as well as cholesterol, have been detected within cartilage—particularly in the superficial zone—and are internalized by chondrocytes via both active and passive transport mechanisms. These findings highlight cartilage as a dynamic metabolic tissue with the capacity to absorb and process extracellular lipids [16].

Endogenous fatty acids are synthesized through de novo lipogenesis (DNL), a pathway in which acetyl‐CoA derived from carbohydrates, amino acids, and other metabolic substrates serves as the fundamental building block. In this process, acetyl‐CoA carboxylase (ACC) catalyzes the conversion of acetyl‐CoA to malonyl‐CoA, which is subsequently utilized by fatty acid synthase (FASN) to generate palmitate—the primary product of DNL and a precursor for the synthesis of diverse fatty acid species [17, 18]. Recent work identified a unique population of lipid‐laden chondrocytes that generate large intracellular lipid vacuoles through ACC1‐ and FASN‐dependent de novo fatty acid synthesis. These cells, termed *lipochondrocytes*, are transcriptionally distinct from adipocytes and lack canonical lipolytic enzymes (ATGL, HSL, and MGL), rendering their lipid stores exceptionally stable [19]. Consistent with this concept, our group previously reported that obesity induces ACC1 upregulation in chondrocytes, suggesting enhanced DNL as a contributor to lipid accumulation in cartilage [15].

Stable isotope tracing using deuterated water (D_2_O) has emerged as a powerful approach for quantifying in vivo lipid synthesis and turnover. This method enables direct measurement of deuterium incorporation into fatty acids, providing quantitative estimates of lipogenic flux across tissues and organs [20, 21]. Applying isotope‐labeling strategies to cartilage offers a unique opportunity to directly assess chondrocyte lipogenesis and determine how it is altered under pathological conditions such as OA.

Despite growing interest in cartilage lipid metabolism, whether articular cartilage possesses the intrinsic capacity to synthesize palmitate through active DNL has not been directly examined. In this study, we applied in vivo D_2_O labeling to quantify palmitate synthesis within cartilage and to define the contribution of ACC1‐dependent DNL to chondrocyte metabolism.

## 2 Methods

### 2.1 Animal Models

Cartilage‐specific ACC1 knockout (*ACC1‐CKO*) mice were generated by crossing *ACC1* ^*flox/flox*^ mice on a C57BL/6J background with *Acan‐Cre* ^*ERT2*^ transgenic mice. To induce Cre‐mediated recombination, tamoxifen (15 mg/mL) was administered intraperitoneally to 1‐month‐old male *ACC1‐CKO (Acan‐Cre* ^*ERT2*^ *; ACC1* ^*flox/flox*^, *n* = *5)* and male WT *(ACC1* ^*flox/flox*^, *n* = *5)* mice once daily for five consecutive days. After induction, the mice were allowed to grow until 3 months of age before being used for experiments. All mice were housed in a pathogen‐free barrier facility under a 12‐h light/dark cycle with ad libitum access to standard rodent chow (ProLab RMH 3000) and water. Animals were allocated to experimental groups based on genotype. All animal procedures were approved by the Institutional Animal Care and Use Committee (IACUC) at Ohio University. To confirm cartilage‐specific ACC1 deletion, immunohistochemical (IHC) staining for ACC1 protein was performed on knee joint sections from WT and ACC1‐CKO mice (*n* = 3 per group). Two tissue sections per animal were stained, and the ratio of ACC1‐positive chondrocytes to total chondrocytes was quantified in a blinded manner and averaged per biological replicate.

### 2.2 Isotope Labeling Protocol

To initiate isotope labeling, a sterile 0.9% (w/v) saline solution was prepared using deuterium oxide (D_2_O) as the solvent. Briefly, 9 g of NaCl was dissolved in 1 L of D_2_O, and the solution was passed through a 0.2 µm non‐pyrogenic filter for sterilization. Three‐month‐old mice received an intraperitoneal (IP) injection of this 0.9% NaCl/D_2_O solution at a dose of 35 µL per gram of body weight using a 1‐mL syringe fitted with a 26‐gauge needle. Following injection, animals were provided with 8% (v/v) D_2_O‐enriched drinking water, prepared by mixing 80 mL of 100% D_2_O with 920 mL of autoclaved regular drinking water obtained from the animal facility. The solution was sterilized through a 0.2 µm non‐pyrogenic filter before use. Experimental groups included male WT and *ACC1‐CKO* mice. Tissue samples were collected at 10‐day and 21‐day labeling time points. A parallel control group received standard drinking water without D_2_O to determine baseline isotopomer distributions and natural isotope abundances.

### 2.3 Serum and Cartilage Tissue Collection, Processing, and Storage

- Mice were anesthetized with isoflurane (Isospire, 99.9%) using a precision vaporizer system (SomnoSuite, Item # SS‐01). Once a deep plane of anesthesia was confirmed, animals were euthanized in accordance with IACUC‐approved protocols.
- Blood collection: Immediately following euthanasia, whole blood was collected *via* cardiac puncture using a 1 mL syringe fitted with a 26‐gauge needle. The collected blood was transferred into 1.5 mL Eppendorf tubes and kept at room temperature for 30 min to allow clot formation.
- Serum isolation and storage: Samples were then centrifuged at 3000 rpm for 25 min at 4°C (degrees Celsius), and the serum layer was carefully separated without disturbing the underlying red blood cell pellet, then transferred to fresh 1.5 mL tubes. The serum volume was measured and recorded for each sample prior to storage. Subsequently, the serum was snap‐frozen in liquid nitrogen and stored at −80°C until further analysis.
- Using a # 11 surgical scalpel, the articular cartilage was gently separated from the tibial plateau and femoral condyles, taking care to avoid contamination from subchondral bone and surrounding soft tissues. Each collected tissue sample from two hind limbs of one mouse weighed approximately 15–25 mg.
- Cartilage pieces were collected and transferred into the pre‐weighed centrifuge tubes. The final weight of the centrifuge tube was recorded to determine the exact mass of the cartilage. The samples were snap‐frozen in liquid nitrogen and stored at −80°C until further analysis.
- To assess tissue purity, Safranin O/Fast Green staining was performed on representative harvested tissue sections collected using this standard dissection protocol (Supplementary Figure S2). While articular cartilage was identifiable by strong Safranin O‐positive proteoglycan‐rich staining, histological examination also revealed the presence of subchondral bone (Fast Green‐positive), reflecting the inherent challenge of isolating pure cartilage from mouse knee joints, where articular cartilage is exceptionally thin (~100–200 µm) and tightly adhered to the underlying bone.

### 2.4 Deuterated Water Enrichment Analysis

To analyze deuterated water enrichment in plasma, 10 µL of plasma was transferred into a new vial. Each sample was mixed with 2 µL of 5 M sodium hydroxide and 4 µL of acetone/acetonitrile solution (5% acetone in 95% acetonitrile). The sodium hydroxide provides a strong basic environment that catalyzes hydrogen–deuterium exchange between body water and acetone, allowing deuterium from D_2_O to be incorporated into the methyl groups of acetone.

Acetonitrile acts as an organic solvent that promotes efficient mixing and prevents protein precipitation, while acetone serves as the exchange substrate that forms deuterium‐labeled acetone.

After brief vortexing and centrifugation, samples were kept at room temperature overnight to allow the isotopic exchange reaction to reach equilibrium. A set of calibration standards containing known D_2_O percentages (0.1, 0.2, 0.4, 0.8, 1.6, 3.2, and 6.4%) was prepared and processed in parallel using the same reaction conditions as the plasma samples.

After the overnight reaction, the samples were extracted with 500 µL of chloroform to separate the deuterium‐labeled acetone from the aqueous phase. Chloroform efficiently dissolves small organic molecules like acetone while removing residual salts and water, providing a clean extract for GC–MS analysis. The organic phase was collected and analyzed using an Agilent 8890 GC coupled to an Agilent 5977 MS system equipped with a DB‐5MS capillary column (30 m long). The instrument was operated in selected ion monitoring (SIM) mode, targeting ions m/z 58, 59, and 60 to detect unlabeled (M_0_) and deuterium‐labeled (M_1_, M_2_) acetone species.

### 2.5 Fatty Acid Deuterium Enrichment Analysis

A volume of 30 µL of plasma and 20 µL of internal standard (C17:0, 2 mM in methanol) were added to a microcentrifuge tube for fatty acid deuterium enrichment analysis. To hydrolyze lipids, 300 µL of 1 M sodium hydroxide in 70% ethanol was added, and samples were incubated at room temperature for 3 h. The alkaline ethanol solution promotes saponification, breaking down complex lipids (such as triglycerides and phospholipids) into free fatty acids, which can then be analyzed by GC–MS.

For cartilage samples, approximately 10 ceramic beads (1 mm) were added for tissue disruption, followed by 20 µL of internal standard (C17:0, 2 mM in methanol) and 300 µL of 1 M sodium hydroxide in 70% ethanol. After eight rounds of bead beating (30 s per cycle, with 30‐s intervals at 4°C), the samples were incubated at room temperature for 3 h to ensure complete lipid hydrolysis.

The saponified samples (both from plasma and cartilage) were extracted separately with 500 µL of chloroform to isolate free fatty acids from the aqueous phase. Chloroform efficiently dissolves lipids and separates them from salts and other polar contaminants. A volume of 100 µL of the chloroform layer from plasma samples and 200 µL from cartilage samples was transferred to new vials.

After drying the organic extracts under gentle air flow, the samples were derivatized with 50 µL of pyridine and 50 µL of BSTFA (N, O‐bis(trimethylsilyl)trifluoroacetamide) containing 1% TMCS (trimethylsilyl chloride). Pyridine serves as both a solvent and a catalyst, while BSTFA reacts with the hydroxyl and carboxyl groups of fatty acids to form trimethylsilyl (TMS) esters, which increase volatility and thermal stability for gas chromatography. The derivatization reaction was carried out at 65°C for 1 h to ensure complete silylation. The resulting fatty acid TMS derivatives were analyzed by GC–MS (Agilent) using a DB‐5MS capillary column.

### 2.6 Data Analysis

The data analysis was performed with MassHunter quantitative analysis (Agilent). The enrichment of deuterated water was analyzed with the linear curve (prepared along with the samples). Lipid enrichment was calculated based on the method developed by Fernandez et. al. [22].

Lipid isotopomer data obtained from GC‐MS were processed using MassHunter Quantitative Analysis software (Agilent). To ensure accurate quantification of deuterium incorporation, the measured isotopomer distributions were corrected for the contribution of naturally occurring heavy isotopes (^13^C, ^2^H, ^15^N, and ^18^O). This correction was performed following the algorithm developed by Fernandez et al., which applies a mathematical correction matrix based on the elemental composition of each fatty acid [22]. This approach estimates the theoretical isotopic pattern expected from natural isotope abundance and subtracts it from the experimental data, yielding the true isotopomer distribution that reflects only deuterium labeling from D_2_O.

Palmitate was used to evaluate DNL. Palmitate concentrations were calculated relative to the internal standard heptadecanoic acid. The newly synthesized palmitate was calculated with total palmitate multiplied by the sum of deuterium labeling (sigma Mn = M1*1 + M2*2 + M3*3), divided by the total replaceable hydrogen (*n* = 22 for palmitate), and then normalized to the plasma volume or tissue weight. Finally, the newly synthesized palmitate was normalized to the D_2_O enrichment in plasma. To further validate the normalization approach, protein concentration was determined by bicinchoninic acid (BCA) assay on parallel cartilage homogenates collected from the same cohort under identical tissue processing conditions as the samples submitted for GC‐MS isotope analysis (standard curve R^2^ = 0.998).

### 2.7 In Vivo DNL Calculation

The rate of newly synthesized fatty acids was determined using deuterium (^2^H, D_2_O) incorporation into palmitate. The fraction of newly formed fatty acids (fractional new synthesis, FNS) was calculated according to the equation:

$$\mathrm{FNS}=\frac{\mathrm{ME}}{N\times P}$$

where *ME* represents the average molar enrichment of palmitate, *P* is the deuterium enrichment of body water measured from plasma samples, and *N* corresponds to the number of hydrogen atoms in palmitate that can be replaced by deuterium during fatty acid synthesis.

The value of *N* was estimated following the relationship described by Lee et al. [23], which relates the isotopic ratio of m_2_ to m_1_ palmitate isotopomers to the enrichment of body water:

$$\frac{m_{2}}{m_{1}}=\frac{\left(N-1\right)}{2}\times \frac{P}{1-P}$$

which can be rearranged to calculate *N* as:

$$N=\left(\frac{\left(m_{2}/m_{1}\right)}{\left(P/(1-P)\right)}\times 2\right)+1$$

The molar amount of newly synthesized fatty acids (MNS) was then obtained by multiplying the FNS by the total concentration of the fatty acid in the tissue sample (nmol/mg).

MNS=FNS×totalfattyacidamount(nmol/mgtissue)

For instance,when *N* was calculated as 21.36, and the corresponding FNS was 0.618, assuming a total palmitate concentration of 2 mmol/mg tissue and a body‐water enrichment (*P*) of 0.045, the estimated amount of newly synthesized palmitate was approximately 1.24 mmol/mg.

During the labeling period, plasma D_2_O enrichment was consistently maintained around 4%–5%, confirming that body water quickly reached and sustained equilibrium with the 8% (v/v) D_2_O provided in drinking water.

### 2.8 Statistical Analysis

All data are presented as mean ± SEM. Each data point represents an individual mouse, with *n* = 5 biological replicates per group unless otherwise indicated. Statistical analyses were performed using GraphPad Prism version 10.4.1. Time‐course FNS data were analyzed by nonlinear regression using a sigmoidal model (four‐parameter logistic fit) to assess labeling kinetics over time. Comparisons between two groups were performed using unpaired two‐tailed Student's *t*‐tests. A *p* value < 0.05 was considered statistically significant. Specific statistical tests, sample sizes, and significance levels are indicated in the corresponding figure legends. Sample sizes (*n* = 5 mice per group) were selected based on prior published D_2_O‐based lipogenic flux studies using comparable experimental designs and analytical approaches [24]. A post‐hoc power analysis was performed on our primary outcome measure, cartilage palmitate abundance at day 10 (Figure 3B). The observed effect size was Cohen's d = 2.027, with WT mean = 7.99 ± 0.41 and KO mean = 5.58 ± 0.63 (SEM). At *n* = 5 per group and α = 0.05 (two‐tailed), the achieved statistical power was 80.1%, meeting the conventional 80% threshold and confirming that *n* = 5 was adequate for detecting our primary significant finding (*p* = 0.0125). We acknowledge that secondary comparisons, including FNS values between genotypes were underpowered at *n* = 5.

## 3 Results

### 3.1 FNS of Palmitate in Serum and Cartilage

First, to validate the labeling workflow, we measured deuterium incorporation into palmitate over a 5‐day D_2_O exposure in wild‐type (WT) mice. FNS was calculated as the fraction of deuterium‐labeled palmitate species relative to the total palmitate pool, corrected for natural isotope abundance. This metric provides an estimate of the fraction of molecules synthesized de novo during the labeling period. It is important to note that in cartilage, FNS values reflect a kinetic rate of ongoing DNL accumulated during the labeling window, rather than fractional replacement of the entire lipid pool, given that cartilage did not reach isotopic equilibrium within the initial labeling period.

As shown in Figure 1A, FNS increased progressively in the serum, with the majority of label incorporation occurring in the first 2 days, after which values approached a plateau. The labeling kinetics fit well to a nonlinear rise‐to‐plateau curve, consistent with steady incorporation of deuterium into fatty acids until pool equilibration. As a contrast, the incorporation of deuterium into fatty acids in the cartilage increased steadily over the labeling period, displaying a continuous upward trajectory without reaching an observable plateau within the experimental timeframe (Figure 1B). Unlike the rapid early rise seen in serum FNS, the kinetics in cartilage exhibited a more gradual, near‐linear accumulation of labeling. This pattern suggests slower turnover dynamics and incomplete equilibration of the fatty acid pool in the matrix‐rich cartilage environment.

**Figure 1:**
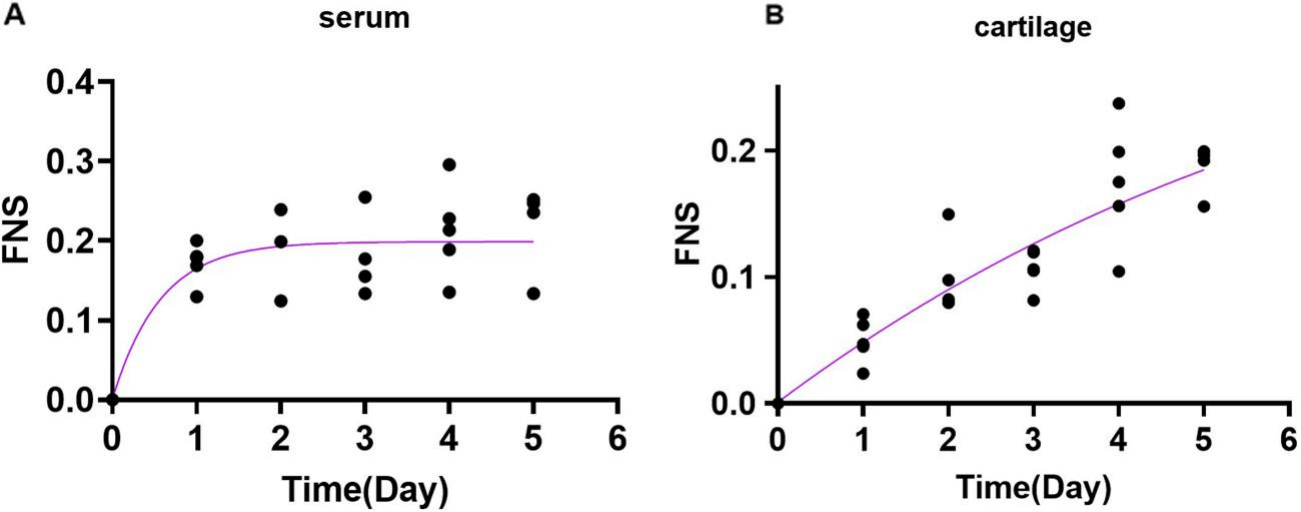
Fractional new synthesis (FNS) of palmitate in serum and cartilage in WT mice during 5 days of D_2_O labeling. (A) Serum FNS rose rapidly within the first 2 days and began to plateau. (B) Cartilage FNS increased steadily over the 5‐day period without reaching a plateau, indicating slower fatty‐acid turnover in cartilage (*n* = 5).

### 3.2 Comparison Between WT and *ACC1‐CKO* Mice

We next compared labeling patterns between WT and *ACC1‐CKO* mice and extended the labeling period to 21 days to ensure isotopic steady state in the cartilage tissue. We first confirmed ACC1 deletion prior to isotope labeling experiments through IHC staining for ACC1 protein. ACC1‐positive chondrocytes were significantly reduced in *ACC1‐CKO* mice compared to WT controls (WT: 0.4000 ± 0.0333 vs. CKO: 0.0623 ± 0.0129; approximately 84.4% reduction; *p* = 0.0060), confirming successful Cre‐mediated recombination in the majority of aggrecan‐expressing chondrocytes (Supplementary Figure S1). Protein concentrations measured by BCA assay also showed comparable protein content between WT (415.4 ± 38.3 µg/µL) and *ACC1‐CKO* (439.6 ± 23.1 µg/µL) groups (Supplementary Table 1).

Both WT and *ACC1‐CKO* mice displayed robust incorporation of D_2_O into palmitate (Figure 2A,B), indicating that the workflow is reproducible and sensitive across groups. Although the overall FNS trajectories did not differ significantly between WT and *ACC1‐CKO* in either the serum or cartilage (Figure 2), analysis of palmitate abundance revealed tissue‐specific differences (Figure 3). Notably, cartilage FNS reached an apparent plateau between day 10 and day 21 in both genotypes (Figure 2B), indicating that isotopic equilibrium of the replaceable cartilage lipid fraction was achieved within the 21‐day labeling window. As in the initial pilot labeling period (Figure 1), the FNS values reported here represent the accumulated kinetic rate of de novo palmitate synthesis over the labeling interval, rather than the fractional replacement of the total cartilage lipid pool; the plateau observed between days 10 and 21 therefore reflects stabilization of this labeling rate within the actively cycling lipid fraction.

**Figure 2:**
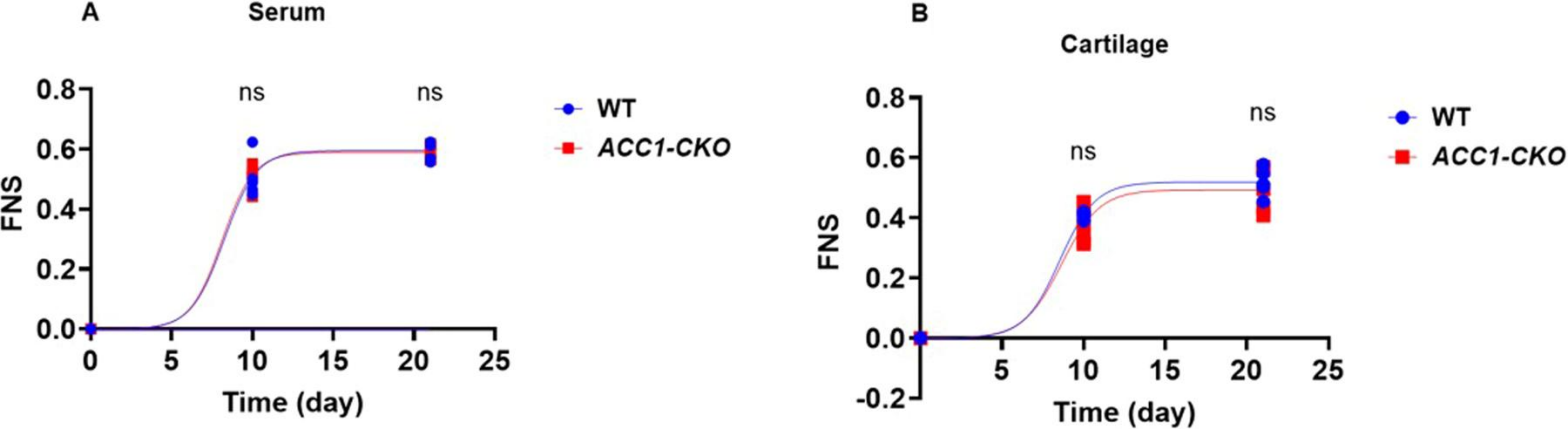
Fractional new synthesis (FNS) of palmitate in serum and cartilage following D_2_O labeling. (A) Time‐course of palmitate FNS in serum from WT and ACC1‐CKO mice following continuous D_2_O administration for up to 21 days. (B) Time‐course of palmitate FNS in cartilage under the same labeling conditions. (ns *p* value > 0.05, *n* = 5, Statistical analysis was determined by two‐tailed Students *t*‐test).

**Figure 3:**
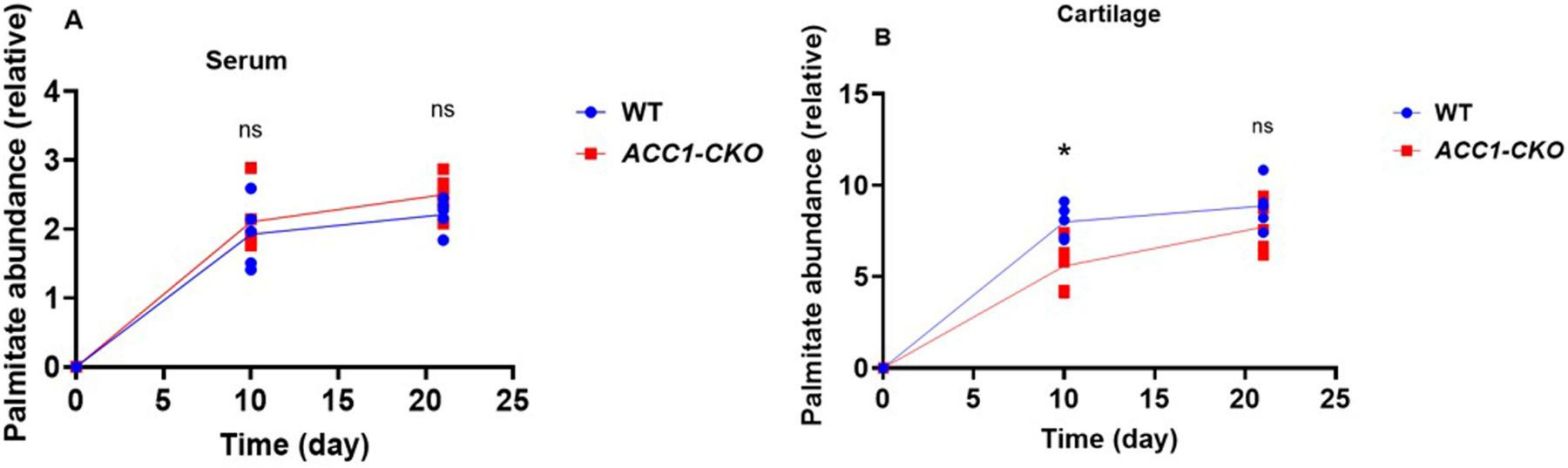
Relative palmitate abundance in WT and ACC1‐CKO mice. (A) *Serum*: Palmitate abundance increased over time in both WT and *ACC1‐CKO* mice with no significant genotype differences. (B) *Cartilage*: Palmitate abundance also rose over time, but *ACC1‐CKO* mice showed a significant reduction compared with WT at day 10. Palmitate levels were quantified by mass spectrometry and normalized to the internal standard (C17:0); measurements were obtained at days 0, 10, and 21 of labeling. (*p value = 0.0125, ns *p* value > 0.05, Data are mean ± SEM, *n* = 5, Statistical analysis was determined by two‐tailed Students *t*‐test).

Relative palmitate abundance, normalized to the internal standard (C17:0), increased progressively over the labeling period in both serum and cartilage, consistent with time‐dependent incorporation of newly synthesized fatty acids (Figure 3A,B). In serum (Figure 3A), palmitate abundance rose rapidly by day 10 and approached a plateau by day 21 in both WT and *ACC1‐CKO* mice. No significant differences were observed between genotypes at any time point, indicating that systemic palmitate pools are largely preserved despite cartilage‐specific deletion of ACC1 and likely reflect contributions from multiple peripheral tissues.

In contrast, cartilage exhibited a distinct, genotype‐dependent pattern (Figure 3B). While palmitate abundance increased over time in both WT and *ACC1‐CKO* cartilage, *ACC1‐CKO* mice displayed a significant reduction in relative palmitate abundance at day 10 compared to WT controls (*p* < 0.05). By day 21, palmitate levels in *ACC1‐CKO* cartilage partially recovered and were no longer significantly different from WT, suggesting compensatory mechanisms or increased contribution from circulating lipid sources at later time points.

Together, these data demonstrate a dissociation between systemic and local lipid metabolism and support a cartilage‐intrinsic role for ACC1 in driving de novo palmitate synthesis, particularly during the early phase of isotope labeling. The transient reduction observed in *ACC1‐CKO* cartilage underscores the importance of ACC1‐mediated lipogenesis in establishing initial fatty acid pools within cartilage, while highlighting the capacity of the tissue to adapt over time through alternative metabolic pathways or lipid uptake.

### 3.3 Isotopomer Distribution Patterns (M0–M4)

To further dissect labeling patterns, we analyzed the distribution of mass isotopomers of palmitate (M0–M4). In this analysis, M0 represents unlabeled molecules, while M1–M4 represent palmitate species with one to four incorporated deuterium atoms. After 10 and 21 days of labeling, both serum and cartilage showed a progressive reduction in M0 species, accompanied by increases in M1–M4 isotopomers. This confirms stepwise incorporation of deuterium into newly synthesized fatty acids.

Isotopomer analysis revealed largely comparable labeling patterns between WT and *ACC1‐CKO* mice across tissues and time points, indicating robust reproducibility of deuterium incorporation and overall consistency of the analytical workflow (Figure 4A–D). In both genotypes, the M0 and low‐mass isotopomers (M1–M2) predominated, reflecting substantial contributions from pre‐existing lipid pools and partial labeling during the experimental window. These baseline distributions were preserved in serum, further supporting the notion that systemic fatty acid pools are relatively insensitive to cartilage‐specific ACC1 deletion.

**Figure 4:**
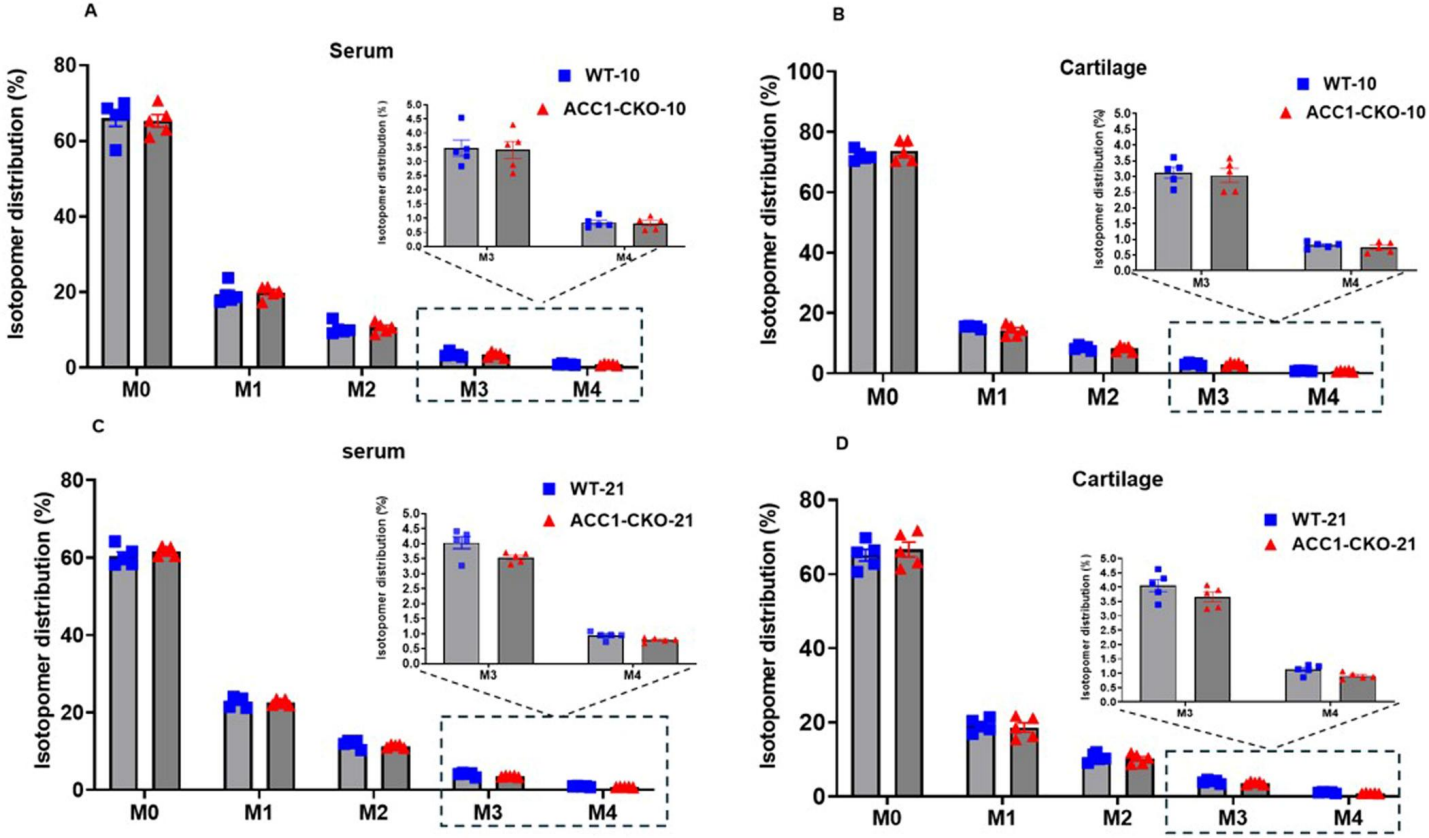
Mass isotopomer distribution (M0–M4) of palmitate in serum and cartilage. WT and *ACC1‐CKO* mice were labeled with D_2_O for 10 (A and B) or 21 (C and D) days. Shifts from M0 toward higher isotopomers confirmed active de novo palmitate synthesis. Insets. No significant differences in isotopomer distribution were detected between genotypes in either tissue at either time point. (ns, not significant. Data are mean ± SEM, *n* = 5. Statistical analysis was determined by two‐tailed Student's *t*‐test).

Together, these findings indicate that ACC1 deletion does not alter the pattern of deuterium incorporation into palmitate isotopomers, suggesting that the labeling mechanism of fatty acid synthesis remains intact in ACC1‐CKO chondrocytes.

### 3.4 Relative Palmitate Pool Size and Dynamics

Normalization to the internal standard enabled separation of isotopic labeling kinetics from changes in absolute lipid abundance, allowing parallel assessment of fatty acid synthesis rates and pool size (Figure 5A,B). In serum (Figure 5A), palmitate abundance increased rapidly during the early labeling phase and approached a plateau at later timepoints in both WT and *ACC1‐CKO* mice. No genotype‐dependent differences were observed, consistent with serum palmitate levels being dominated by dietary input and contributions from multiple peripheral tissues rather than cartilage‐intrinsic synthesis.

**Figure 5:**
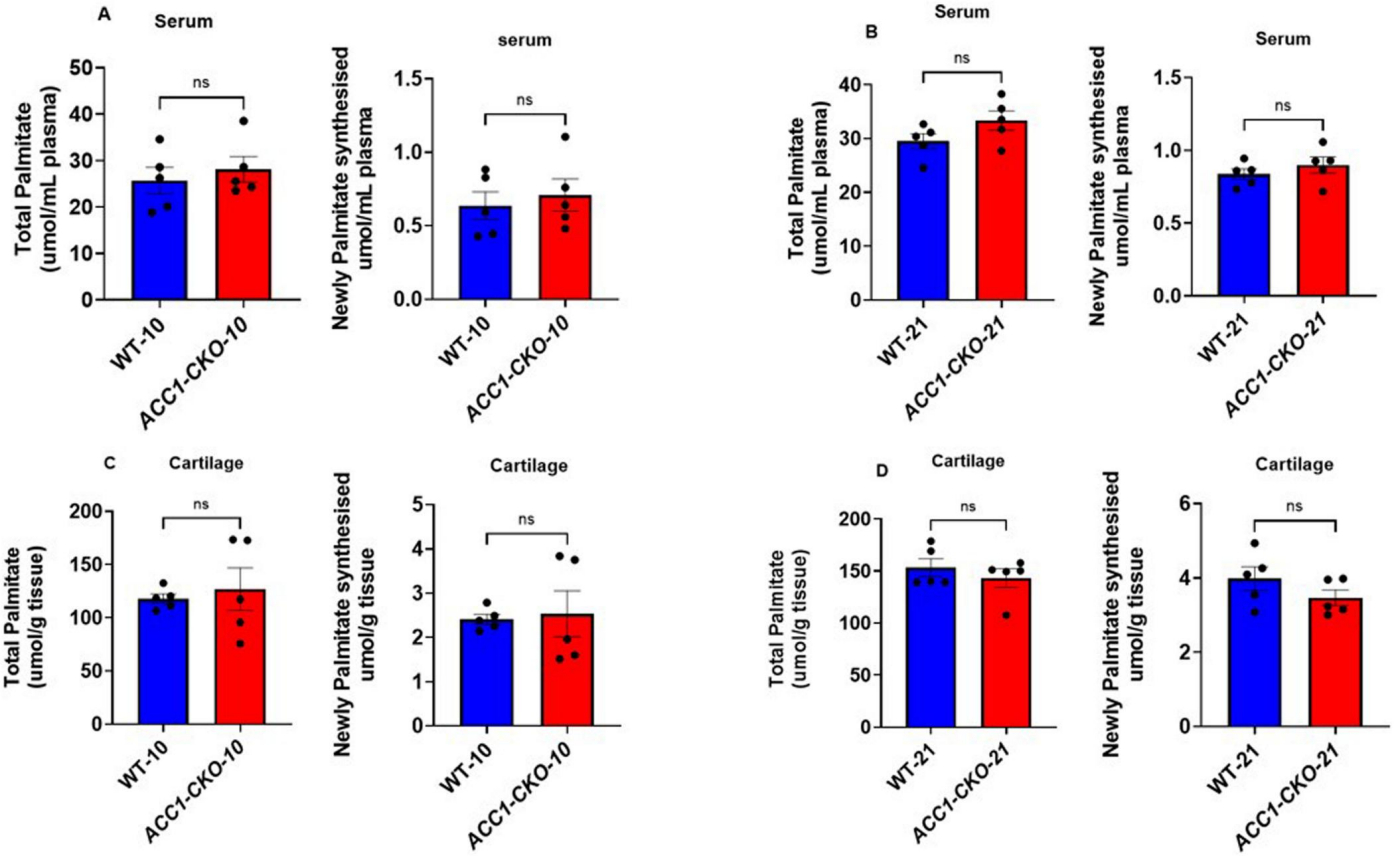
De novo synthesized palmitate and total palmitate levels in serum and cartilage of WT and ACC1‐CKO mice after D_2_O labeling. (A and B) *Serum*: At both day 10 (A) and day 21 (B), total palmitate levels and newly synthesized palmitate did not differ significantly between WT and *ACC1‐CKO* mice. (C and D) *Cartilage*: Similarly, at day 10 (C) and day 21 (D), total and de novo–synthesized palmitate levels were comparable between genotypes. Palmitate abundance was quantified by mass spectrometry, and newly synthesized palmitate was calculated from deuterium incorporation after correction for natural isotope abundance. ns, not significant. (Data are mean ± SEM, *n* = 5. Statistical analysis was determined by two‐tailed Student's *t*‐test).

In contrast, cartilage exhibited a distinct pattern (Figure 5B). Although palmitate abundance increased over time in both groups, *ACC1‐CKO* mice displayed a significant reduction in normalized palmitate levels at day 10 compared to WT controls. Importantly, this decrease in total palmitate abundance occurred despite comparable FNS between genotypes at the same time point, indicating that ACC1 deletion did not abolish the capacity for palmitate synthesis per se. Rather, *ACC1‐CKO* cartilage maintained a smaller steady‐state palmitate pool, suggesting reduced overall lipid accumulation or increased turnover.

Together, these findings highlight a functional distinction between lipid synthesis kinetics and pool size regulation in cartilage. While both WT and *ACC1‐CKO* chondrocytes are capable of incorporating newly synthesized palmitate, ACC1 activity appears to be required to sustain normal lipid abundance during the early labeling phase. These results further demonstrate that D_2_O tracing, when coupled with internal standard normalization, provides a powerful means to simultaneously resolve dynamic synthesis rates and tissue‐specific changes in lipid pool size.

## 4 Discussion

DNL is a fundamental anabolic pathway through which cells synthesize fatty acids from small‐molecule precursors, primarily acetyl‐CoA [25]. While glucose‐derived carbon is the dominant substrate for systemic DNL, alternative metabolites such as acetate, lactate, fructose, and branched‐chain amino acids can also contribute in a tissue‐specific manner [26, 27, 28]. Dysregulated DNL has been implicated in a wide range of pathological conditions, including cancer [29], cardiometabolic disease [30], and metabolic syndrome [31, 32]. Central to this pathway is ACC, which catalyzes the formation of malonyl‐CoA, the committed substrate for FASN–mediated palmitate production. Although DNL has been extensively studied in highly metabolic tissues such as liver and adipose [20, 24, 27, 33, 34], its contribution to cartilage metabolism has remained largely unexplored.

Here, we applied in vivo deuterium oxide (D_2_O) labeling to directly quantify fatty‐acid synthesis in cartilage. Stable isotope tracing offers a powerful and physiologically relevant approach to assess metabolic flux by monitoring the incorporation of non‐radioactive isotopes into newly synthesized molecules [35, 36, 37]. In contrast to short‐term tracer infusion methods, D_2_O labeling rapidly equilibrates with body water and provides a uniform precursor pool, enabling accurate and reproducible measurement of biosynthetic rates over extended periods. Consistent with prior work in other tissues [38, 39], we observed efficient and progressive incorporation of deuterium into palmitate, validating D_2_O labeling as a robust method for assessing cartilage lipid synthesis in vivo.

Recent studies have demonstrated that cartilage is metabolically active and capable of engaging lipid biosynthetic pathways. Evidence from genetic and metabolic analyses indicates that dysregulated DNL, driven by altered acetyl‐CoA metabolism, contributes to cartilage degeneration under osteoarthritic conditions [40]. Obesity‐associated metabolic cues have been shown to activate ACC–dependent DNL in chondrocytes, leading to intracellular lipid accumulation that can be mitigated by inhibition of this pathway [15]. Extending these observations, recent work has identified a distinct population of lipid‐filled chondrocytes in mammalian cartilage that rely almost exclusively on DNL for lipid vacuole formation, independent of circulating fatty acid uptake. In this context, lipid synthesis is not merely a pathological byproduct but a tightly regulated metabolic program that contributes to cartilage structure and biomechanical properties, highlighting a fundamental role for endogenous lipid biosynthesis in cartilage biology [19].

Kinetic analysis revealed distinct labeling patterns between serum and cartilage. Palmitate FNS in serum increased rapidly and approached a plateau within days, reflecting the high turnover and metabolic accessibility of circulating lipid pools. In contrast, cartilage exhibited a slower, near‐linear increase in palmitate labeling that did not fully plateau within the early labeling window. This delayed equilibration is consistent with the dense extracellular matrix and limited vascular access of cartilage [41, 42], which likely constrain lipid exchange and turnover. These findings highlight fundamental tissue‐specific differences in lipid dynamics and emphasize the importance of measuring cartilage metabolism directly rather than inferring it from systemic lipid pools. While the initial 5‐day pilot experiment showed near‐linear FNS accumulation without plateau (Figure 1B), extending the labeling period to 21 days revealed that an apparent plateau was achieved between days 10 and 21 (Figure 2B), confirming that the 21‐day window captures equilibration of the replaceable lipid fraction.

Importantly, the detection of substantial deuterium incorporation into cartilage palmitate provides direct in vivo evidence that chondrocytes possess intrinsic lipogenic activity. This observation challenges the prevailing view that cartilage relies predominantly on exogenous lipid uptake from synovial fluid or surrounding tissues [43]. While prior studies have demonstrated that chondrocytes can internalize fatty acids and cholesterol [16, 44, 45], our data establish that endogenous fatty‐acid synthesis contributes measurably to the cartilage lipid pool under physiological conditions. Thus, cartilage should be considered a metabolically active tissue capable of sustaining lipid renewal through autonomous biosynthetic pathways.

This interpretation is further supported by a recent characterization of lipochondrocytes, a distinct chondrocyte subpopulation whose lipid content is generated mainly via DNL and which exhibits marked downregulation of the enzymatic machinery required for lipid catabolism and exogenous lipid uptake, thereby sequestering these lipid stores from systemic metabolic influence [19]. Although this population was characterized primarily in elastic cartilage, an analogous metabolically quiescent lipid compartment, if present in articular chondrocytes, would be expected to remain largely unlabeled under D_2_O exposure. Accordingly, the plateau observed in cartilage FNS between days 10 and 21 (Figure 2B) more plausibly reflects equilibration of the actively cycling lipid fraction rather than complete turnover of the total cartilage lipid pool.

Using cartilage‐specific ACC1 knockout mice, we further demonstrate that ACC1 contributes to maintaining the cartilage palmitate pool. Although steady‐state FNS values were broadly similar between genotypes, ACC1 deficiency was associated with reduced palmitate abundance in cartilage at early labeling time points and modest decreases in higher‐order isotopomers, suggesting diminished lipogenic flux. These findings indicate that ACC1‐dependent malonyl‐CoA production supports endogenous fatty‐acid synthesis in chondrocytes, particularly during periods of active lipid turnover. The persistence of labeling in ACC1‐deficient cartilage may reflect compensatory mechanisms, incomplete recombination, or contributions from alternative metabolic pathways, underscoring the metabolic flexibility of chondrocytes.

In mammalian cells, acetyl‐CoA functions as a key metabolic intermediate that connects major pathways such as glycolysis, the TCA cycle, amino acid metabolism, gluconeogenesis, and fatty acid synthesis. ACCs catalyze the conversion of acetyl‐CoA to malonyl‐CoA, a critical step that supplies the substrate required by FASN for the production of long‐chain saturated fatty acids during DNL [46, 47]. DNL is initiated when ATP‐citrate lyase (ACLY) converts citrate into acetyl‐CoA. This acetyl‐CoA is then carboxylated by acetyl‐CoA carboxylase (ACC1/acaca) to form malonyl‐CoA, which serves as the building block for FASN to elongate carbon chains and generate palmitate, a 16‐carbon saturated fatty acid that represents the primary product of this pathway [48, 49]. By catalyzing the formation of malonyl‐CoA, ACC1 not only supplies substrate for de novo fatty acid synthesis but also modulates fatty acid oxidation through malonyl‐CoA–dependent inhibition of mitochondrial fatty acid entry [50]. Genetic and pharmacological studies across metabolically active tissues, including liver, adipocytes, immune cells, endothelial cells, and pancreatic endocrine cells, demonstrate that ACC1 activity is essential for maintaining appropriate lipid composition, cellular energetics, and functional adaptation. Disruption of ACC1 consistently leads to reduced lipogenesis, altered phospholipid and acyl‐CoA pools, and downstream defects in cell growth, signaling, or secretory capacity, underscoring ACC1 as a core regulator of fatty acid homeostasis rather than a context‐limited lipogenic enzyme [51, 52, 53, 54, 55, 56].

Methodologically, this work establishes a practical and quantitative framework for studying cartilage lipid metabolism in vivo. By combining D_2_O labeling with GC–MS‐based isotopomer analysis, this approach enables simultaneous assessment of lipid synthesis kinetics, pool size, and pathway flux without perturbing physiological conditions. Beyond palmitate, this workflow can be extended to interrogate broader lipid classes or applied to disease models, aging, and metabolic stress conditions relevant to OA.

The primary goal of this study was to establish and validate a quantitative in vivo workflow for measuring DNL in articular cartilage. The *ACC1‐CKO* model was included to demonstrate the sensitivity of this approach in detecting genotype‐dependent differences in lipogenesis, rather than to fully define the phenotypic effects of ACC1 deletion. The functional impact of ACC1 deficiency on cartilage integrity has been examined in separate HFD studies in our laboratory, including Safranin O histology and OARSI scoring. In addition, an important limitation of this study is that the harvested mouse cartilage samples were not completely free of calcified cartilage or subchondral bone. Safranin O/Fast Green staining of representative harvested tissues confirmed the presence of articular cartilage as well as subchondral bone regions (Supplementary Figure S2), indicating that these samples should be interpreted as cartilage‐enriched joint tissues rather than pure hyaline cartilage. Although this may contribute to the total lipid signal measured by GC–MS, the same standardized dissection protocol was applied across all groups, and ACC1 deletion was driven by *Acan‐Cre* ^*ERT2*^. Thus, the observed genotype‐dependent reduction in palmitate abundance likely represents a conservative estimate of ACC1‐dependent lipogenesis in cartilage. Another limitation of this study is that palmitate abundance was normalized to cartilage tissue mass rather than DNA or hydroxyproline content. Because cartilage hydration, cellularity, and matrix composition may vary among animals, DNA‐ or hydroxyproline‐based normalization would provide additional information regarding cell‐normalized or matrix‐normalized lipid abundance. The original GC–MS samples were fully consumed during lipid extraction, preventing retrospective analysis. However, BCA protein quantification of parallel cartilage homogenates from the same cohort showed comparable total protein content between WT and ACC1‐CKO samples, suggesting that major differences in tissue input or gross sample composition are unlikely to fully explain the observed genotype‐dependent differences. Future studies using larger tissue inputs or optimized parallel sampling will incorporate DNA and/or hydroxyproline normalization.

In summary, our findings demonstrate that cartilage is capable of de novo fatty‐acid synthesis in vivo and that ACC1 plays a functional role in regulating cartilage lipid homeostasis. This work provides both conceptual and technical foundations for future studies investigating how dysregulated lipogenesis contributes to cartilage degeneration and OA pathogenesis. By enabling direct measurement of chondrocyte lipid synthesis, D_2_O‐based isotope tracing offers a valuable tool for dissecting metabolic mechanisms within this traditionally understudied tissue.

## Author Contributions

**Shadi Moradi:** experiments, data collection, data analysis, manuscript writing and editing; **Anupama Binoy:** experiments, manuscript writing and editing; **Shouan Zhu:** conceptualization, project supervision, funding acquisition, manuscript writing and editing.

## Ethics Statement

All animal studies have been approved by the Ohio University Institutional Animal Care and Use Committee (IACUC) (Protocol number: 00000043).

## Conflicts of Interest

The authors declare no conflicts of interest.

## Supporting information


Supporting File

## Data Availability

The data that support the findings of this study are available from the corresponding author upon reasonable request.
