# Supplementary material for: Stable Isotope Labeling to Track De Novo Fatty Acid Synthesis in Mouse Cartilage‐Enriched Joint Tissue In Vivo: A Practical Workflow
Source: J Orthop Res. 2026 Oct 4;44(10):e70290. doi: 10.1002/jor.70290 (PMC13635674; doi:10.1002/jor.70290)
Supplement: Supplementary file 1 [file JOR-44-0-s001.docx]

**SUPPLEMENTARY FIGURES**

WT

*ACC1-CKO*

***Supplementary Figure S1.*** ACC1 immunohistochemical staining in WT and ACC1-CKO articular cartilage. Representative IHC images show robust ACC1 protein expression in WT cartilage (left), indicated by brown cytoplasmic staining, whereas ACC1-CKO cartilage shows markedly reduced staining (right). Quantification shows the percentage of ACC1-positive chondrocytes among total chondrocytes. WT: 0.4000 ± 0.0333 vs. *ACC1-CKO*: 0.0623 ± 0.0129; approximately 84.4% reduction; p = 0.0060; n = 3 per group. ** p < 0.01. Statistical analysis was determined by two-tailed Student's t-test. Scale bars = 20 µm.

***Supplementary Figure S2.*** Safranin O/Fast Green staining of representative harvested tibial plateau tissue collected using the standard dissection protocol. Red-orange Safranin O-positive areas indicate proteoglycan-rich hyaline articular cartilage. Fast Green-positive regions indicate subchondral bone. Scale bars = 200 µm.

***Supplementary Table 1*. Protein concentrations of parallel cartilage tissue homogenates from WT and ACC1-CKO mice used in the stable isotope labeling study.**

| **Sample ID** | **Genotype** | **OD (avg)** | **OD − Blank** | **Protein conc. (µg/µL)** | **Volume for 100 µg (µL)** |
| --- | --- | --- | --- | --- | --- |
| WT1 | WT | 0.289 | 0.177 | 442.5 | 22.6 |
| WT2 | WT | 0.267 | 0.155 | 388.3 | 25.8 |
| KO1 | ACC1-CKO | 0.294 | 0.182 | 455.8 | 21.9 |
| KO2 | ACC1-CKO | 0.281 | 0.169 | 423.3 | 23.6 |

*Protein concentrations were determined by bicinchoninic acid (BCA) assay (standard curve R² = 0.998) on parallel cartilage homogenates collected from the same cohort under identical tissue processing conditions as samples submitted for GC-MS isotope analysis. OD, absorbance at 562 nm; Blank, mean absorbance of blank wells (0.112). WT, wild-type; ACC1-CKO, cartilage-specific ACC1 conditional knockout (Aggrecan-Cre^ERT2^ × ACC1ᶠˡᵒˣ/ᶠˡᵒˣ).*
